# The identification of miRNA and mRNA expression profiles associated with pediatric atypical teratoid/rhabdoid tumor

**DOI:** 10.1186/s12885-022-09549-6

**Published:** 2022-05-06

**Authors:** Xinke Xu, Hongyao Yuan, Junping Pan, Wei Chen, Cheng Chen, Yang Li, Fangcheng Li

**Affiliations:** 1 Department of Neurosurgery, The First Affiliated Hospital of Jinan University, Guangzhou, China; 2 Department of Neurosurgery, Guangzhou Women and Children’s Medical Center, Guangzhou, China

**Keywords:** Atypical teratoid, MRNA, MicroRNA, Expression profiles, Immunocyte infiltration

## Abstract

**Background:**

Atypical teratoid/rhabdoid tumor (AT/RT) is a malignant pediatric tumor of the central nervous system (CNS) with high recurrence and low survival rates that is often misdiagnosed. MicroRNAs (miRNAs) are involved in the tumorigenesis of numerous pediatric cancers, but their roles in AT/RT remain unclear.

**Methods:**

In this study, we used miRNA sequencing and gene expression microarrays from patient tissue to study both the miRNAome and transcriptome traits of AT/RT.

**Results:**

Our findings demonstrate that 5 miRNAs were up-regulated, 16 miRNAs were down-regulated, 179 mRNAs were up-regulated and 402 mRNAs were down-regulated in AT/RT. qPCR revealed that hsa-miR-17-5p and MAP7 mRNA were the most significantly differentially expressed miRNA and mRNA in AT/RT tissues. Furthermore, the results from analyses using the miRTarBase database identified MAP7 mRNA as a target gene of hsa-miR-17-5p.

**Conclusions:**

Our findings suggest that the dysregulation of hsa-miR-17-5p may be a pivotal event in AT/RT and miRNAs that may represent potential therapeutic targets and diagnostic biomarkers.

**Supplementary Information:**

The online version contains supplementary material available at 10.1186/s12885-022-09549-6.

## Background

Atypical teratoid/rhabdoid tumor is the most common malignant embryonal central nervous system tumor in children below 12 months of age; however, the incidence of AT/RT is not yet known and its incidence rate decreases with age [1, 2]. AT/RT was first identified as one of the embryo tumors that represent approximately 1–2% of pediatric intracranial tumors [3]. Because of the lack of clinical manifestation and radiography characteristics, the early clinical diagnosis of AT/RT remains challenging [4, 5]. The treatment options for AT/RT currently include surgical resection, chemotherapy and radiotherapy [6–8]. However, the prognosis for pediatric patients with AT/RT is still dismal, with a median survival of 15.4 months. Studies have shown that the majority of AT/RT patients show genomic mutations in *SMARCB1* (also known as *INI1*) [9]. However, the precise pathogenesis of this disease is unclear. The identification of novel therapeutics based on the specific mechanism of AT/RT carcinogenesis is therefore critical.

Recent studies have shown that microRNAs (miRNAs) play a vital role in CNS tumorigenesis. MiRNAs, a subtype of small non-coding RNAs, regulate gene expression through recognizing and binding to seed sequence–matching sites in the 3' untranslated regions of target mRNAs [10–12]. MiRNAs are involved in the pathogenesis of human malignant tumors and function as oncogenes or tumor suppressors, depending on their downstream targets [13–15]. Previous studies have identified abnormal miRNA levels in patients with tumors in the CNS, indicating that miRNAs may play a key role in CNS tumor development [16]. However, knowledge of the miRNA expression profile of AT/RT patients is still limited.

Previous studies revealed that some miRNAs are deregulated in AT/RT, suggesting a role of miRNAs as oncogenes or tumor suppressors [17]. The miRNA expression signatures were found to be associated with bio-molecular and prognostic characteristics in AT/RT. Roles of miRNAs in the regulation of AT/RT, through destabilization or translational repression of target mRNAs, have been widely investigated. However, a comprehensive analysis of the miRNA expression profile in pediatric AT/RT has not yet been conducted.

In this study, we determined the miRNA expression profiles of pediatric AT/RT tumors by analyzing public datasets. Our results revealed that 5 miRNAs were significantly upregulated and 16 miRNAs were significantly downregulated in tumor tissue. Furthermore, we found that abnormal hsa-miR-17-5p expression may play key functions in the tumorigenesis in AT/RT and may represent potential targets for clinical treatment.

## Methods

### Differential expression analysis of GEO datasets

The datasets generated and/or analysed during the current study are available in the GSE42656 (https://www.ncbi.nlm.nih.gov/geo/query/acc.cgi?acc=GSE42656) and GSE42657 (https://www.ncbi.nlm.nih.gov/geo/query/acc.cgi?acc=GSE42657) repository. were processed by edgeR package in RStudio (version 3.5.0), with a significant cutoff |log2FC|> 2 and *P*-value < 0.01 [18]. The gene expression profile GSE42656 contains eight control and five AT/RT patients. The miRNA expression dataset is derived from GSE42657, which includes seven control and five tumor tissues. Detailed information of the datasets is listed in Table 1.

**Table 1:** Datasets for AT/RT

Datasets	Platform	Description	Controls	Tumors
GSE42656	GPL6947	gene	**8**	**5**
GSE42657	GPL8179	miRNA	**7**	**5**

### Functional analysis

Based on results from the differential expression analysis, we identified the related signaling pathways using Gene Ontology (GO) enrichment analysis. GO terms have three different modules: biological process (BP), molecular function (MF), and cellular component (CC). KEGG pathway analysis was used to identify the significant pathways for dysregulated mRNAs [19–21]. GO and KEGG analysis were both used in cluster profiler package in R studio [22]. The *P*-value was calculated for each enriched function and/or pathway.

### Immunocyte infiltration annotation

We used the CIBERSORT approach to identify inflammatory gene expression signatures in silico to identify the characteristics of the immune response in AT/RT. CIBERSORT is a computational framework for high-throughput characterization of immune cells [23].

### miRNA-mRNA pair analysis

We used miRTarBase to predict the target genes of the differentially expressed miRNAs (http://mirtarbase.mbc.nctu.edu.tw/) [24]. Differentially expressed genes (DEGs) were extracted and the putative miRNA-mRNA regulatory network was constructed using Cytoscape software (version 3.7.0). To validate the miRNA-mRNA network, we calculated the Pearson values and depicted the correlograms through R software. We evaluated the negative correlation between the key miRNA and target expression.

### Reverse transcription quantitative real-time PCR (RT-qPCR)

We used a gene chip to analyze the gene expression profiles. cDNA fragments were purified with a PCR extraction kit (RR037A, Takara, China) following the manufacturer’s instructions and then enriched by RT-PCR. Total RNA was extracted using TRIzol reagent (Life Technologies, USA) and quantified using Thermo Nanodrop 2000. RNA (0.5 μg) was subjected to reverse transcription using the Script cDNA Synthesis Kit (RR037A, Takara, China). miRNA and mRNA primer sequences are listed in Tables 2 and 3.

**Table 2:** miRNA primers

Gene	Species	Sequence
hsa-miR-17-5p	*Homo sapiens*	CAAAGTGCTTACAGTGCAGGTAG
hsa-miR-18a-5p	*Homo sapiens*	TAAGGTGCATCTAGTGCAGATAG
hsa-miR-488-5p	*Homo sapiens*	CCCAGATAATGGCACTCTCAA
hsa-miR-128-3p	*Homo sapiens*	TCACAGTGAACCGGTCTCTTT
hsa-miR-495-3p	*Homo sapiens*	AAACAAACATGGTGCACTTCTT
hsa-miR-668-3p	*Homo sapiens*	TGTCACTCGGCTCGGCCCACTAC
hsa-miR-874-3p	*Homo sapiens*	CTGCCCTGGCCCGAGGGACCGA
U6-F	*Homo sapiens*	CTCGCTTCGGCAGCACA
U6-R	*Homo sapiens*	AACGCTTCACGAATTTGCGT
Universal-R	*Homo sapiens*	GCTGTCAACGATACGCTACG

**Table 3:** mRNA primers

Gene	Species	Sequences
*CCND1*	*Homo sapiens*	TGAGGGACGCTTTGTCTGTC
*CCND1*	*Homo sapiens*	TGAGGGACGCTTTGTCTGTC
*CDC20*	*Homo sapiens*	AATGTGTGGCCTAGTGCTCC
*CDC20*	*Homo sapiens*	AGCACACATTCCAGATGCGA
*CDK1*	*Homo sapiens*	GGCTCTGATTGGCTGCTTTG
*CDK1*	*Homo sapiens*	ATGGCTACCACTTGACCTGT
*PTTG1*	*Homo sapiens*	TAACTGGACCAACGGCAACT
*PTTG1*	*Homo sapiens*	AGAGCTAAACAGCGGAACAGT
*PPP3R1*	*Homo sapiens*	CGGGTGTTAGGCCAGCTATT
*PPP3R1*	*Homo sapiens*	AGCTCTTGGCAGTAGCAATGA
*CDCA5*	*Homo sapiens*	CTGAGCAGTTTGATCTCCTGGT
*CDCA5*	*Homo sapiens*	CTCAAAGGCAGACAGTCCTCA
*PRKCB*	*Homo sapiens*	GACCAAACACCCAGGCAAAC
*PRKCB*	*Homo sapiens*	GATGGCGGGTGAAAAATCGG
*HDAC1*	*Homo sapiens*	TGCTAAAGTATCACCAGAGGGT
*HDAC1*	*Homo sapiens*	GGAGCGGGTAGTTAACAGCA
*MAP7*	*Homo sapiens*	TGCCAAGTGGCTGGTACTAT
*MAP7*	*Homo sapiens*	GGAATTGGCCTTGCATTGGT
*DPYSL2*	*Homo sapiens*	AGATCCAACTTTGCCGCTT
*DPYSL2*	*Homo sapiens*	CGTCTGCCAGTCCCTAAGT
*CD47*	*Homo sapiens*	ACCTCCTAGGAATAACTGAAGTG
*CD47*	*Homo sapiens*	GGGTCTCATAGGTGACAACCA
*GAPDH*	*Homo sapiens*	AACGGATTTGGTCGTATTGGG
*GAPDH*	*Homo sapiens*	CCTGGAAGATGGTGATGGGAT

### Statistical analysis

Statistical analyses are presented as means ± SD. Comparison between two groups was performed using two-tailed Student’s t tests, and two-way ANOVAs with general linear model procedures using a univariate approach were applied for more than two groups. All statistical analyses were performed with GraphPad 8 Prism software (San Diego, CA, USA). *P* < 0.05 was considered statistically significant.

## Results

### Differential expression profiles for pediatric AT/RT

**Fig. 1:**
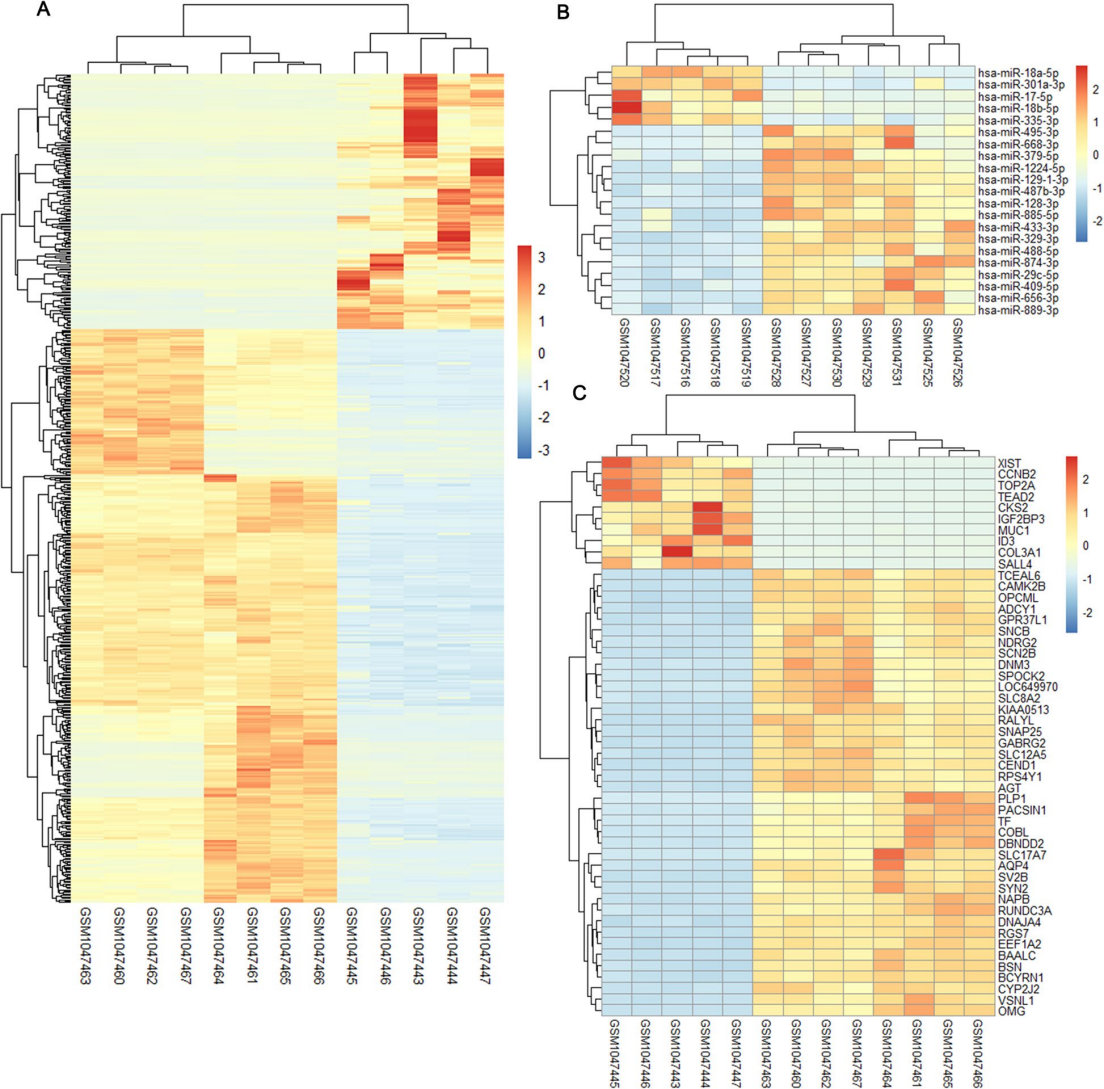
Differential gene expression analysis of pediatric atypical teratoid tumors. **A** Heat map depicting gene expression from 13 AT/RT cases and normal brain (columns; ordered automatically by hierarchical clustering). A gradient “heat spectrum” appears at the right; red indicates increased expression, whereas blue denotes decreased levels. **B** Heat map illustrating the expression of 50 mRNAs. **C** Heat map illustrating the expression of 21 differentially expressed miRNAs (fold change > 2 and *P*-value < 0.01)

Using |log fold change|> 2 and *P*-value < 0.01 as a threshold, we identified a total of 581 DEGs in the tumor group compared with the control group. Among the DEGs, 179 were up-regulated and 402 were down-regulated (Fig. 1A, Supplementary Information.). In addition, 21 differentially expressed miRNAs (DEmiRNAs) were identified, including 5 up-regulated DEmiRNAs and 16 down-regulated DEmiRNAs (Table 4, Fig. 1B–C).

**Table 4:** DEmiRNAs

miRNA	logFC	*P-*value	dysregulated
hsa-miR-129–1-3p	-4.59055	2.23E-25	down
hsa-miR-128-3p	-4.46518	4.64E-22	down
hsa-miR-656-3p	-3.58948	5.81E-10	down
hsa-miR-329-3p	-3.45779	1.42E-11	down
hsa-miR-1224-5p	-3.22655	1.05E-07	down
hsa-miR-668-3p	-3.13363	3.91E-07	down
hsa-miR-488-5p	-3.11733	1.15E-09	down
hsa-miR-29c-5p	-3.0582	2.44E-10	down
hsa-miR-379-5p	-3.03907	1.70E-07	down
hsa-miR-885-5p	-2.88115	2.84E-05	down
hsa-miR-433-3p	-2.60679	5.77E-05	down
hsa-miR-874-3p	-2.4917	1.26E-07	down
hsa-miR-409-5p	-2.44799	5.69E-06	down
hsa-miR-487b-3p	-2.39918	1.40E-09	down
hsa-miR-495-3p	-2.20978	6.25E-06	down
hsa-miR-889-3p	-2.10228	9.37E-08	down
hsa-miR-301a-3p	2.157525	8.66E-07	up
hsa-miR-18a-5p	2.319174	3.73E-10	up
hsa-miR-335-3p	2.374372	3.30E-06	up
hsa-miR-18b-5p	3.442157	7.63E-10	up
hsa-miR-17-5p	3.53329	7.91E-08	up

### GO enrichment analysis for DEGs

**Fig. 2:**
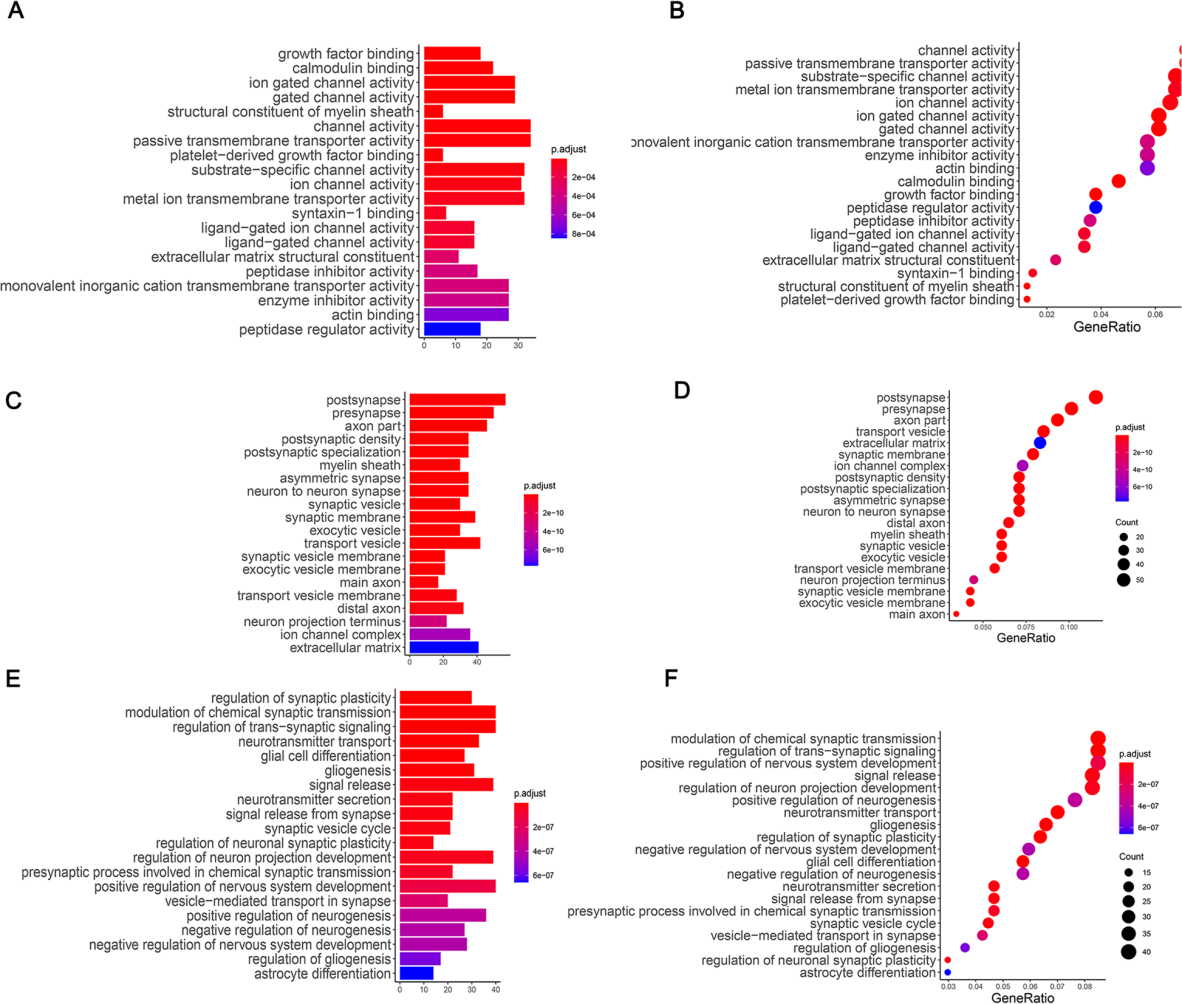
GO enrichment analysis for DEGs in pediatric atypical teratoid tumors. **A**, **C**, **E** Barplots show the top 20 enrichment terms of CC, MF, and BP, respectively. Each bar represents a term, and the length represents the number of genes enriched. **B**, **D**, **F** Dotplots represent the top 20 enrichment results of CC, MF, and BP, respectively. The size of each point represents the number of genes enriched; the color represents the degree of enrichment

To identify the biological characteristics and signaling pathways involved in the pathogenesis of AT/RT, we next used Clusterprofile in R package to enrich DEGs. The enrichment results of the top 20 genes from the CC, MF and BP categories are shown in Fig. 2. The results indicated that many of the DEGs are closely involved in the formation of synapses. Molecular functions analysis indicated that DEGs were involved in binding to specific molecules, such as growth factor binding, calmodulin binding, and activity of passive membrane transporters. The DEGs were also involved in several critical biological progresses including the regulation of synaptic plasticity, modulation of chemical synaptic transmission, and transportation and secretion of the neurotransmitters, which are all involved in the regulation of nervous system plasticity.

### KEGG enrichment analysis for DEGs and the immune infiltration correlation of the expression profile

**Fig. 3:**
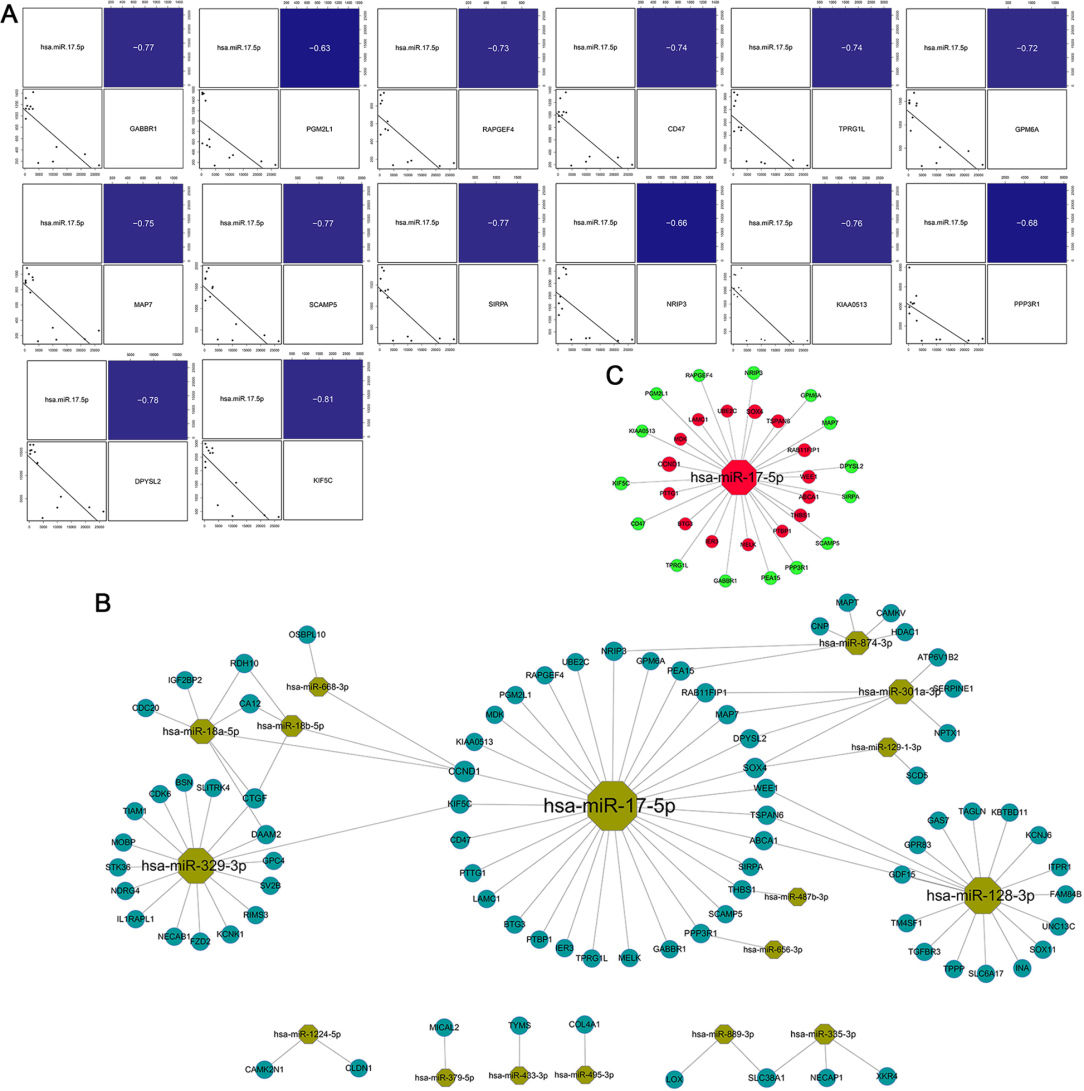
KEGG enrichment analysis for DEGs and the immune infiltration correlation of the expression profile in pediatric AT/RT. **A** Barplot shows the top 20 enrichment results from KEGG. The length represents the number of genes enriched. **B** Dotplot presents the top 20 enrichment results from KEGG. The size of each point represents the number of genes enriched. **C** Bar charts summarize immune cell subset proportions against AT/RT *p*-value by study

The KEGG signaling pathway results are shown in Fig. 3. DEGs are highly involved in synaptic function and neurotransmitter transmission. The top enriched pathways include the regulation of “Synaptic vesicle cycle”, “GABAergic synapse” and “Glutamatergic synapse,” which are consistent with the results of GO enrichment, indicating that impaired synaptogenesis and synaptic dysfunction could contribute to the formation and clinical manifestation of AT/RT. DEGs were also shown to modulate the “cAMP signaling pathway,” which could affect cell differentiation.

We also analyzed the correlation between the expression profile and immune infiltration pathways to identify the association between immune cell types and AT. The proportions of certain immune cells such as memory T cells, resting dendritic cells, neutrophils, and neutrophils were relatively lower in tumor tissues compared with levels in normal tissues. B cells, activated NK cells, and T follicular helper cells showed no difference between tumor tissues and normal tissues.

### Construction of the AT/RT-associated miRNA-mRNA correlation and network

**Fig. 4:**
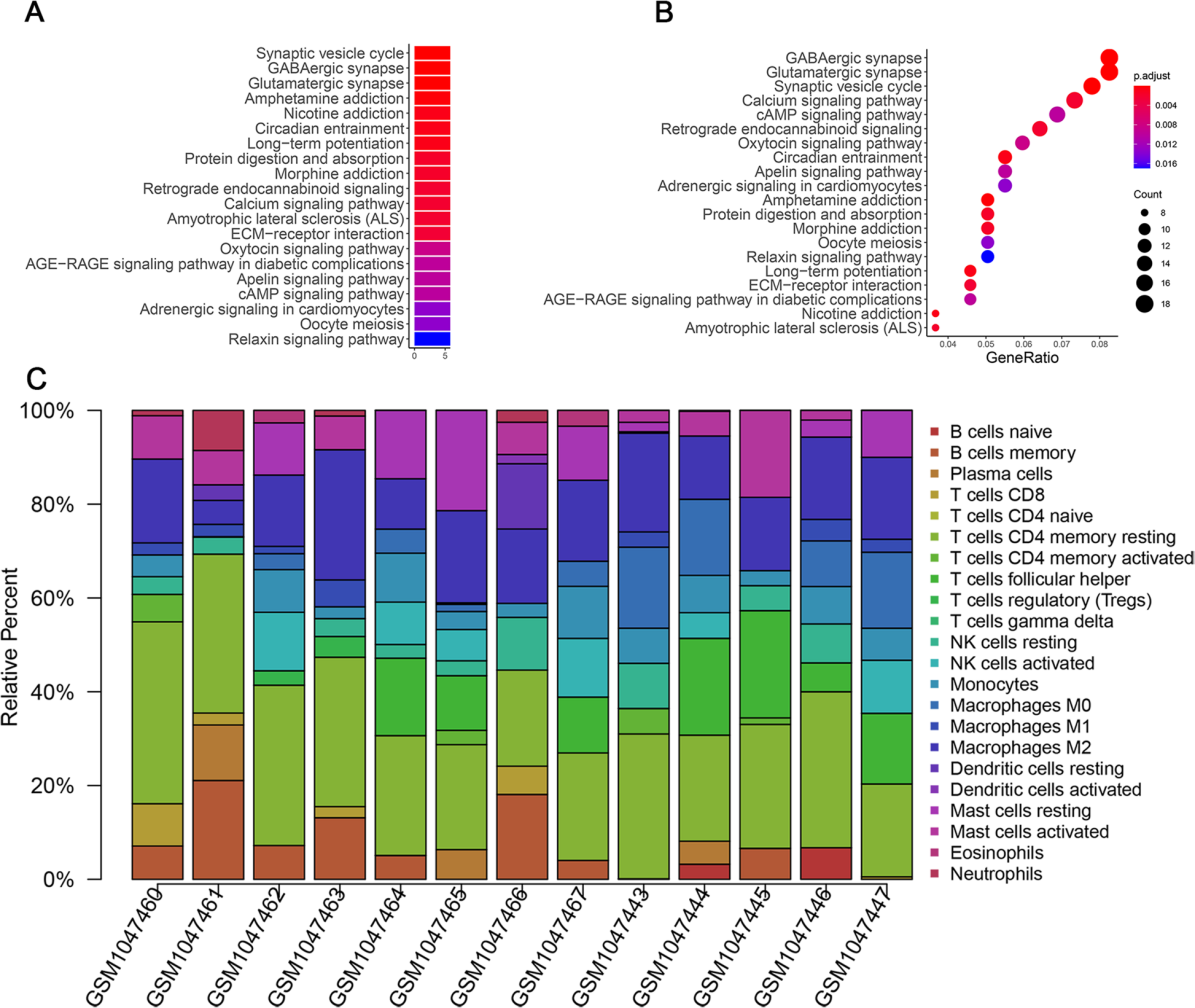
Correlation analysis and network of AT/RT-associated miRNA-mRNA. **A** The correlation analysis of miR-17A-5p and 14 downregulated mRNAs by Pearson coefficient. **B** The subnetworks revealed the molecular pathways that were altered by miR-17a-5p. **C** The co-expression network of differentially expressed miRNAs and mRNAs was constructed

To clarify the potential roles of significantly dysregulated miRNAs and to further explore miRNA-mRNA regulatory mechanisms in AT/RT, we identified the potential targets of DEmiRNAs and the genes that were inversely co-expressed with DEmiRNAs using the previously shown gene expression profile. The 581 DEmRNAs and 21 mature DEmiRNAs were analyzed using the miRTarBase database (http://mirtarbase.mbc.nctu.edu.tw/). A total of 17 DEmiRNAs were found to negatively regulate at least one of the targets in DEmRNAs. Detailed information for each miRNA-mRNA targeting pair is shown in Table 5. The co-expression network of DEmiRNAs and DEmRNAs was constructed and visualized using Cytoscape software; the results are shown in Fig. 4. miR-17a-5p appeared to play the central role in the DEG network; therefore, miR-17A-5p was selected for further analysis.

**Table 5:** Co-expression information of DEmiRNAs and DEmRNAs

DEmiRNA	Targets (DEmRNAs)
hsa-miR-1224-5p	CAMK2N1, CLDN1
hsa-miR-128-3p	WEE1, INA, UNC13C, KCNJ6, GAS7, GPR83, TSPAN6, ITPR1, TAGLN, TM4SF1, SOX11, ABCA1, TGFBR3, FAM84B, TPPP, SLC6A17, KBTBD11, GDF15
hsa-miR-129–1-3p	SOX4, SCD5
hsa-miR-17-5p	CCND1, THBS1, WEE1, SIRPA, SOX4, UBE2C, MDK, KIF5C, PTBP1, GPM6A, DPYSL2, PTTG1, TPRG1L, KIAA0513, SCAMP5, RAPGEF4, NRIP3, MAP7, RAB11FIP1, BTG3, MELK, TSPAN6, PEA15, PPP3R1, PGM2L1, LAMC1, IER3, GABBR1, CD47, ABCA1
hsa-miR-18a-5p	IGF2BP2, CTGF, CA12, DAAM2, CDC20, RDH10, CCND1
hsa-miR-18b-5p	CTGF, RDH10, CA12, CCND1
hsa-miR-301a-3p	SERPINE1, NPTX1, SOX4, ATP6V1B2, RAB11FIP1, DPYSL2, MAP7
hsa-miR-329-3p	TIAM1, KCNK1, CTGF, SV2B, KIF5C, MOBP, NECAB1, DAAM2, GPC4, FZD2, NDRG4, SLITRK4, CDK6, STK36, IL1RAPL1, BSN, RIMS3
hsa-miR-335-3p	NECAP1, XKR4, SLC38A1
hsa-miR-379-5p	MICAL2, MICAL2
hsa-miR-433-3p	TYMS
hsa-miR-487b-3p	THBS1
hsa-miR-495-3p	COL4A1
hsa-miR-656-3p	PPP3R1
hsa-miR-668-3p	OSBPL10, CCND1
hsa-miR-874-3p	CNP, HDAC1, NRIP3, MAPT, CAMKV, PEA15
hsa-miR-889-3p	SLC38A1, LOX

We next examined the regulatory relationship of miR-17a-5p. The subnetworks shown in Fig. 4 revealed the molecular pathways that were altered by miR-17a-5p. There were 15 mRNAs downregulated by miR-17a-5p. In addition, correlation analysis by Pearson coefficient revealed that KIF5C and DPYSL2 had the highest correlation with miR-17a-5p (Fig. 4, Table 6).

**Table 6:** Correlation analysis of target genes of miR-17a-5p

Gene	r^2^	*P*-value
*KIF5C*	-0.8132933	0.0007215
*DPYSL2*	-0.7761295	0.001813395
*PEA15*	-0.7679643	0.002171028
*SIRPA*	-0.7668815	0.002222304
*SCAMP5*	-0.7661486	0.002257544
*KIAA0513*	-0.7638543	0.002370676
*MAP7*	-0.7522732	0.003010815
*CD47*	-0.7433326	0.003590814
*TPRG1L*	-0.7379651	0.003978361
*RAPGEF4*	-0.7284424	0.004744666
*GPM6A*	-0.7216386	0.005358348
*PPP3R1*	-0.6832816	0.010037493
*NRIP3*	-0.6627694	0.01355686
*TSPAN6*	0.2707255	0.370982354
*ABCA1*	0.3972861	0.178873767
*WEE1*	0.5946464	0.032071943
*BTG3*	0.6999231	0.007731892
*MELK*	0.7468654	0.003352146
*PTTG1*	0.8412606	0.000312887
*CCND1*	0.8464124	0.0002637
*MDK*	0.8483379	0.000246983
*UBE2C*	0.8598805	0.000163567

### Validation of related miRNA expression levels in AT/RT using qRT-PCR

**Fig. 5:**
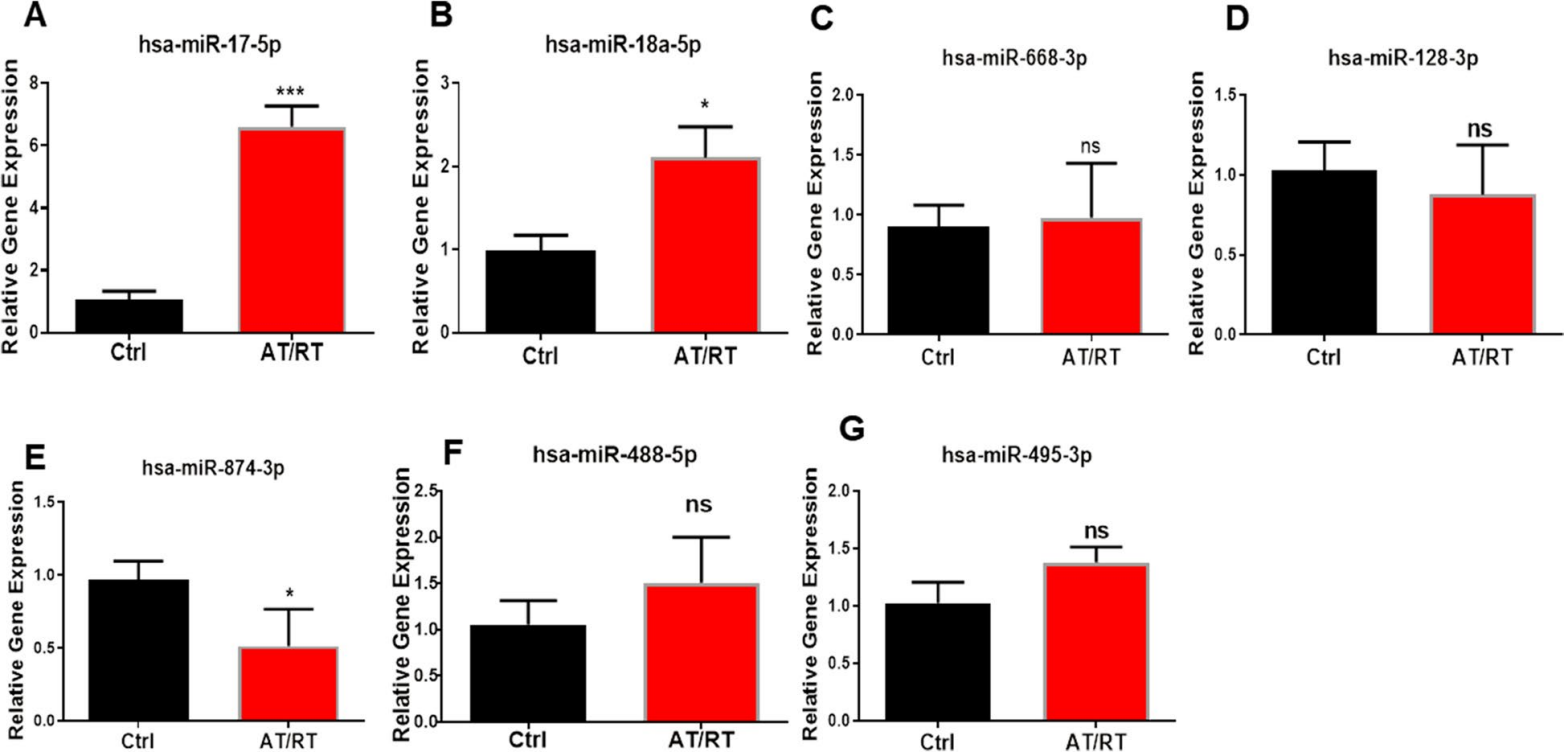
The related miRNA expression level using quantitative real-time PCR in AT/RT. **A–G** The relative expression levels of hsa-miR-17-5p, has-miR-18a-5p, hsa-miR-488-5p, hsa-miR-128-3p, hsa-miR-495-3p, hsa-miR-668-3p, and hsa-miR-874-3p (^*^P < 0.05, compared with control; ^**^P < 0.01, compared with control; ^***^P < 0.001 compared with control)

Previous studies have shown that miRNAs play a vital role in tumor progression in AT/RT. We next evaluated the performance of the seven candidate miRNAs (hsa-miR-17-5p, has-miR-18a-5p, hsa-miR-488-5p, hsa-miR-128-3p, hsa-miR-495-3p, hsa-miR-668-3p, hsa-miR-874-3p) in diagnosing AT/RT. The data demonstrated higher expression of miR-17-5p and miR-18a-5p in AT/RT compared expressions in with normal brain tissues by qPCR (Fig. 5). In addition, the expression of miR-874-3p was lower in AT/RT compared with levels in normal brain tissues.

### Verification for related mRNA expression levels using qRT-PCR

**Fig. 6:**
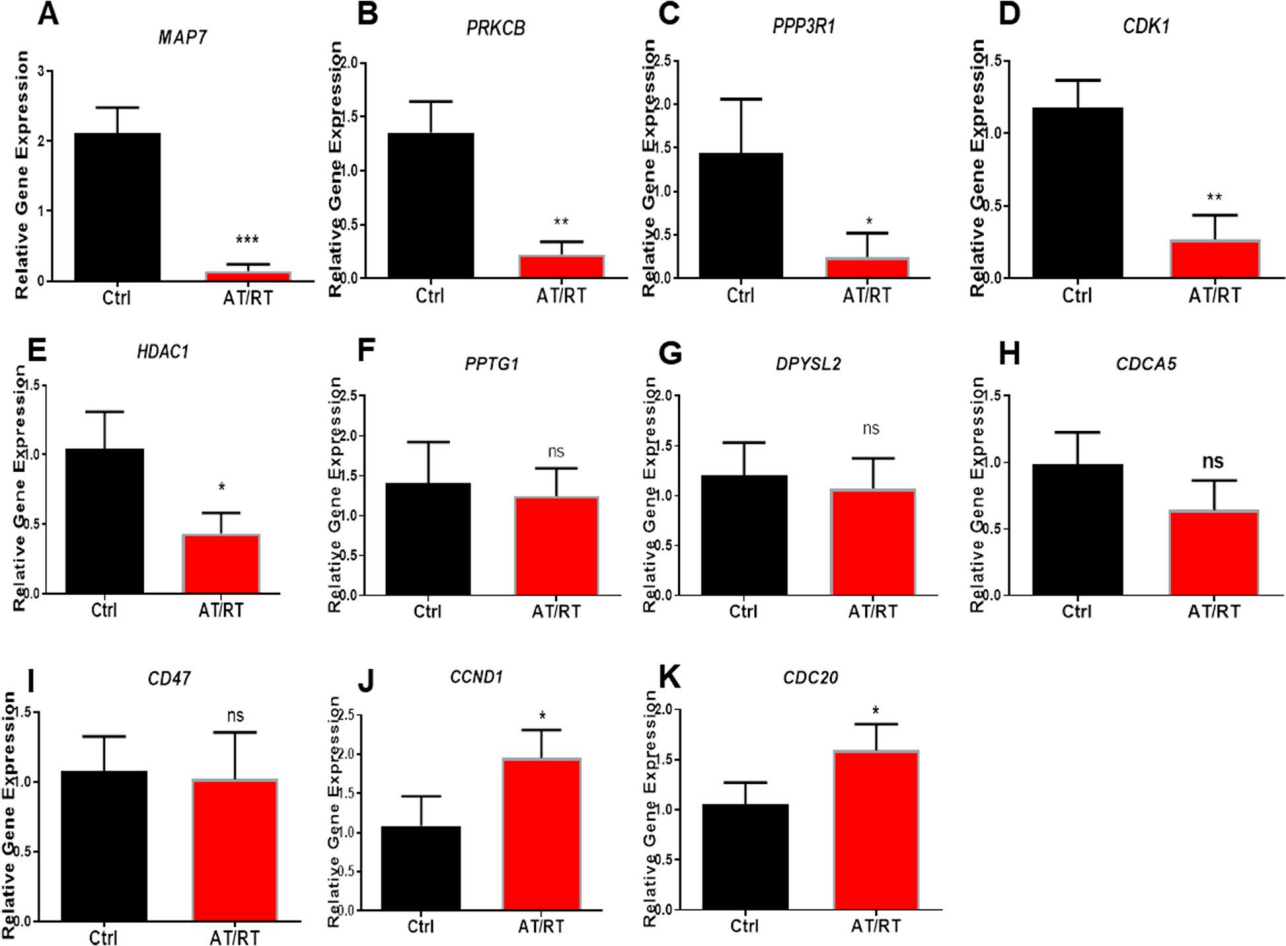
Quantitative real-time PCR of related mRNAs in AT/RT. **A–K** The relative expression levels of MAP7, PRKCB, PPP3R1, CDK1, HDAC1, PPTG1, DPYSL2, CDCA5, CD47, CCND1 and CDC20 mRNAs (^*^P < 0.05, compared with control; ^**^P < 0.01, compared with control; ^***^P < 0.001 compared with control)

To investigate the potential function and underlying mechanism of miR-17-5p in AT/RT, we used bioinformatics algorithms and mRNA profiling from AT/RT patients to identify potential target genes of miR-17-5p. The binding of a miRNA to its target mRNA can induce translational silencing or degradation, leading to inhibition or enhancement of gene expression. Various studies have performed expression profiling to identify the roles of miRNAs in AT/RT. Our results indicated that MAP7, PRKCB, CDK1, PPP3R1, CCND1, HDAC1 and CDC20 mRNAs were differentially expressed in AT/RT (Fig. 6). Notably, our study showed that MAP7 plays an important regulatory role in AT/RT.

## Discussion

AT/RT is an aggressive pediatric tumor of the CNS. Because of the limited available treatments and poor prognosis of AT/RT, there is an urgent need to identify novel therapeutic targets and develop innovative treatment strategies for this disease [25–27]. Mutations and/or deletions of the *SMARCB1* (BAF47/INI1/SNF5) gene are hallmarks of AT/RT tumors, and so far no other recurrent genetic abnormalities have been identified [2, 28]. Previous studies showed that HMGA2, LIN28, RPL5, RPL10 and SUN2 are crucial regulators in AT/RT [29, 30]. However, the precise molecular mechanism of AT/RT remains largely unknown.

miRNAs play crucial roles in regulating gene expression at the transcriptional, post-transcriptional and epigenetic levels. Previous studies have established that miRNAs participate in a wide variety of biological processes including genomic imprinting, cell cycle, cell differentiation, invasion and migration [31–34]. Hsiehet et al. showed that miR-221/222 represents a promising new target in AT/RT [30]. Our multi-omics analysis identified five upregulated miRNAs (hsa-miR-301a-3p, hsa-miR-18a-5p, hsa-miR-335-3p, hsa-miR-18b-5p, hsa-miR-17-5p) and 16 downregulated miRNAs (hsa-miR-129–1-3p, hsa-miR-128-3p, hsa-miR-656-3p, hsa-miR-329-3p, hsa-miR-1224-5p, hsa-miR-668-3p, hsa-miR-488-5p, hsa-miR-29c-5p, etc.) in AT/RT. Hsa-miR-129–1-3p was the most-downregulated in AT/RT while hsa-miR-17-5p was the most up-regulated miRNA in AT/RT.

We further found that 179 mRNAs were up-regulated and 402 mRNAs were down-regulated, which could be the result of the dysregulated miRNA networks in AT/RT, as miRNAs regulate the levels and functions of their target mRNAs. GO analyses revealed that these mRNAs are involved in critical pathways such as the regulation of synaptic plasticity, modulation of chemical synaptic transmission, neurotransmitter transportation and secretion. KEGG pathway analysis showed that “Synaptic vesicle cycle,” “GABAergic synapse” and “Glutamatergic synapse” were related to the DEGs, which is consistent with GO enrichment analysis. These findings suggest that altered synaptogenesis and synaptic dysfunction could contribute to the formation and clinical manifestation of AT/RT. Additionally, DEGs were involved in the canonical pathways such as cAMP signaling pathway, which may contribute to the stemness of the AT/RT tumor cells.

Several recent studies have analyzed the influence of the host immune system on cancer prognosis [35]. We performed analyses using CIBERSORT, a computational method for high-throughput characterization of different types of immune cells in complex tissues. Our results demonstrated there was no difference in immune-related cells in AT/RT.

MiRTarBase database is a database that predict targets for miRNAs [36]. Seventeen DEmiRNAs were found to have at least one negatively regulated miRNA-mRNA pair in the DEmRNAs. Notably, over 30 mRNAs were predicted to be regulated by hsa-miR-17-5p. To further probe the negative correlations between hsa-miR-17-5p and its target mRNAs, we calculated the Pearson values using R software. A total of 15 mRNAs were negatively correlated with hsa-miR-17-5p. In addition to the protein–protein interaction networks constructed between DEmiRNAs and DEmRNAs, we also further verified the expression of hsa-miR-17-5p, hsa-miR-18a-5p, hsa-miR-488-5p, hsa-miR-128-3p, hsa-miR-495-3p, hsa-miR-668-3p, and hsa-miR-874-3p using qPCR [37]. These results further demonstrated the importance of hsa-miR-17-5p in AT/RT.

Zeng et al. previously reported that miRNA-17-5p expression is upregulated in glioblastoma and is a potential marker for the proneural subtype [38]. However, the mechanisms by which miRNA-17-5p expression regulates tumorigenesis are not well elucidated. We screened and identified possible targets of miRNA-17-5p and the results suggested that CCND1, THBS1, WEE1, SIRPA, SOX4, UBE2C, MDK, KIF5C, PTBP1, GPM6A, DPYSL2, PTTG1, TPRG1L, KIAA0513, SCAMP5, RAPGEF4, NRIP3, MAP7, RAB11FIP1, BTG3, MELK, TSPAN6, PEA15, PPP3R1, PGM2L1, LAMC1, IER3, GABBR1, CD47, and ABCA1 genes may play important roles in the pathogenesis of AT/RT. qPCR experiments verified that the expressions of MAP7, CDK1, PPP3R1, PRKC1, CCND1 and HDAC1 genes were indeed altered in AT/RT tumor tissue. Interestingly, *CCND1*, which encodes a crucial regulator of the cell cycle [39], was upregulated in AT/RT. We hypothesize that miRNA-17-5p promotes tumorigenesis in AT/RT by promoting *CCND1* expression and cell cycle entry and progression. In addition, we reported that MAP7 mRNA showed the greatest down-regulation in AT/RT among all identified mRNAs. Together, these studies point to a potential role of miR-17-5p in AT/RT tumorigenesis.

This study has several limitations Future studies are required to determine the effect of miRNA-17-5p in AT/RT cell lines. RNA-seq and brain imaging using large-scale sample sizes would fully represent AT/RT tumorigenesis. Additional studies on the mechanism related to miRNA-17-5p will provide a better understanding of AT/RT therapy.

## Conclusions

The results of this study indicate that upregulated/downregulated miRNAs and mRNAs have potential clinical value as prognostic biomarkers in AT/RT, in particular showing great potential as prognostic molecular markers. We demonstrated that miRNA-17-5p promotes tumorigenesis in AT/RT by promoting CCND1 expression and cell cycle entry and is associated with disease progression. To the best of our knowledge, this is the first study to find that the miRNA-17-5p/*CCND1* axis regulates AT/RT. Further large scale cohort studies focusing on these miRNAs are recommended to verify the clinical utility of these markers individually and/or in combination. These findings provide a better understanding of the pathogenesis and development of AT/RT and may be an important implication for future therapy of the AT/RT.

## Supplementary Information

**Additional file 1.**

## Data Availability

The datasets generated and/or analysed during the current study are available in the GSE42656 (https://www.ncbi.nlm.nih.gov/geo/query/acc.cgi?acc=GSE42656) and GSE42657 (https://www.ncbi.nlm.nih.gov/geo/query/acc.cgi?acc=GSE42657) repository.

## Abbreviations

AT/RT: Atypical teratoid/rhabdoid tumor
CNS: Central nervous system
qPCR: Quantitative Real-time PCR
KEEG: Kyoto Encyclopedia of Genes and Genomes
GO: Gene Ontology
BP: Biological process
MF: Molecular function
CC: Cellular component
DEGs: Differentially Expressed Genes
RT-qPCR: Reverse transcription quantitative Real-time PCR
DEmiRNAs: Differentially expressed miRNAs
